# GLP-1-based therapeutics for cardiorenal protection in metabolic diseases

**DOI:** 10.1093/ndt/gfaf110

**Published:** 2025-06-25

**Authors:** Ellen M Apperloo, Hiddo J L Heerspink, Daniël H van Raalte, Marcel H A Muskiet

**Affiliations:** Department of Clinical Pharmacy and Pharmacology, University of Groningen, University Medical Center Groningen, Groningen, The Netherlands; Department of Clinical Pharmacy and Pharmacology, University of Groningen, University Medical Center Groningen, Groningen, The Netherlands; Diabetes Center, Department of Internal Medicine, Amsterdam University Medical Centers, location VUMC, Amsterdam, The Netherlands; Diabetes Center, Department of Internal Medicine, Amsterdam University Medical Centers, location VUMC, Amsterdam, The Netherlands

**Keywords:** cardiovascular disease, GLP-1, kidney disease, obesity, type 2 diabetes

## Abstract

Over the last decade, significant progress has been made in cardiorenal protection for metabolic diseases such as type 2 diabetes (T2D) and obesity. With an expanding range of pharmacological options and continuously evolving guidelines, glucagon-like peptide-1 receptor agonists (GLP-1RAs) have garnered substantial clinical and societal attention for their role in T2D and weight management. GLP-1RAs have consistently demonstrated robust HbA1c- and body weight–reducing efficacy in clinical and real-world studies. In addition, mounting data established their cardiorenal benefits beyond glycaemic control in select high-risk populations. In T2D, GLP-1RAs have been shown to improve both hard cardiovascular and, more recently, relevant kidney outcomes. Meanwhile, in individuals with obesity but without T2D, semaglutide (at a higher dose than in T2D) reduces body weight by up to 15% and lowers the risk of major adverse cardiovascular events by 20%. The success of GLP-1-based therapy fuelled the development of new single molecules that combine GLP-1R agonism with activation of other entero-pancreatic hormone receptors [e.g. glucose-dependent insulinotropic polypeptide (GIP), glucagon and amylin] aiming to achieve complementary and potentially synergistic effects. These next-generation GLP-1-based therapeutics for metabolic diseases, either already available or approaching clinical approval, appear to enhance metabolic and weight-reducing efficacy compared with existing GLP-1RAs. An example is tirzepatide, a dual GLP-1/GIP receptor agonist, which has been approved for both T2D and obesity management, demonstrating up to 22.5% weight loss in phase 3 trials. This review explores the landscape of current and emerging GLP-1-based therapies, their efficacy in managing hyperglycaemia and body weight, recent evidence supporting their cardiorenal benefits and clinical implications of these advancements.

## INTRODUCTION

The umbrella term ‘metabolic diseases’ encompasses a spectrum of non-communicable diseases, including obesity, type 2 diabetes (T2D), dyslipidaemia and metabolic dysfunction-associated steatotic liver disease (MASLD). These share common metabolic dysregulation, such as insulin resistance, glucose and lipid dysmetabolism and chronic low-grade inflammation. Their prevalence is rising rapidly, often coexisting and contributing to excess disability, morbidity and mortality from cardiovascular diseases (CVDs), chronic kidney disease (CKD) and certain cancers [1].

**Figure 1:**
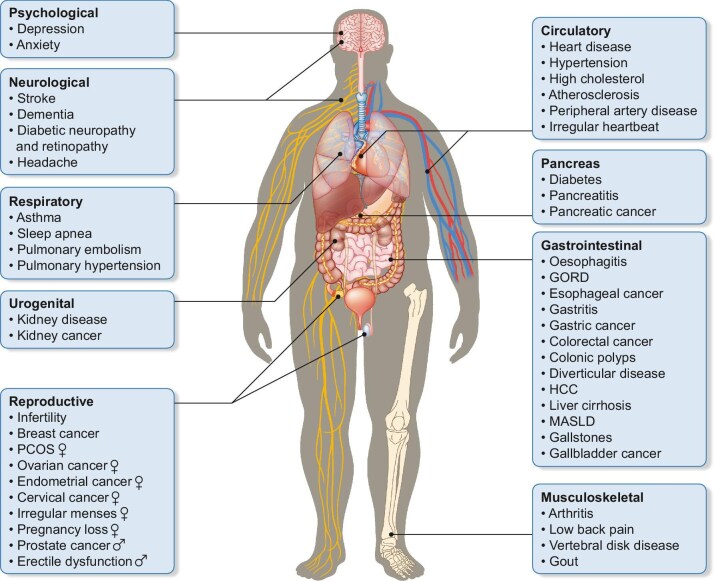
Complications associated with obesity. Overview of obesity-related comorbidities affecting multiple organ systems. Specific conditions are listed as representative examples, highlighting the broad range of health complications associated with obesity.

Managing excess body fat is seen as a central public health challenge [2], with obesity driving the T2D pandemic, responsible for approximately half of new T2D cases [3]. This trend places unprecedented strain on healthcare systems and increases associated medical expenses. High body mass index (BMI) accounts for >50% of global T2D disability-adjusted life years, which rose by 24.3% between 1990 and 2021 [4]. Additionally, obesity is linked to >200 other complications [5], including CVD, heart failure (HF), CKD, sleep apnoea, osteoarthritis and depression (Fig. 1). Consequently, weight loss of >5–10% has now become a key treatment goal in overweight/obese people, with and without diabetes, as it provides substantial metabolic and health benefits. In T2D, recognition that double-digit weight loss has substantial benefits (e.g. T2D remission) further shifted guidelines to prioritize weight management, alongside CV risk reduction and glycaemic control [6].

Lifestyle interventions, including diet, exercise and behavioural changes, remain the cornerstone of weight management, offering multiple health benefits. However, even the most intensive approaches typically achieve only up to 10% average weight loss, with ≈80% of lost weight regained within 5 years [7]. Therefore, current guidelines recommend considering anti-obesity medications [e.g. glucagon-like peptide-1 receptor agonists (GLP-1RAs)] and metabolic surgery alongside lifestyle changes. These interventions produce greater and more sustained weight loss in eligible patients and may provide additional metabolic and cardiorenal benefits. Simultaneously, new classes of cardiorenal medications, some originally approved for glycaemic control, have rapidly gained traction for their ability to reduce CVD, CKD and mortality, independent of haemoglobin A1c (HbA1c) and weight loss. As a result, guidelines in diabetes, cardiology and nephrology now recommend sodium–glucose co-transporter 2 inhibitors (SGLT2is), finerenone and GLP-1RAs in metabolic diseases and high cardiorenal risk.

Advances in drug development are shifting treatment goals from traditional targets such as HbA1c for T2D, weight for obesity and blood pressure (BP) with renin–angiotensin–aldosterone system (RAAS) blockers for CKD towards personalized approaches that prioritize body weight and reduction of major complications in metabolic diseases. Despite decreasing T2D-related mortality in recent decades, residual risk for CVD or renal failure remains high. This review highlights the cardiorenal benefits of current GLP-1RAs in T2D and obesity, explores next-generation GLP-1 formulations and examines potential pharmacologic interactions with other cardiorenal therapies with proven efficacy, such as SGLT2is and finerenone.

## GLP-1RA FOR T2D AND OBESITY

### The incretin effect as a treatment target

The ‘incretin-effect’ describes the physiological phenomenon whereby an oral glucose load elicits a greater insulin secretory response than an equivalent intravenous glucose administration due to insulinotropic signals from the gastrointestinal tract [8]. This effect is mediated by gut-derived hormones, including but possibly not limited to glucose-dependent insulinotropic polypeptide (GIP) and GLP-1. The importance of incretin action for normal glucose homeostasis is well established, accounting for ≈70% of postprandial insulin secretion in healthy individuals [8]. However, the incretin effect is uniformly defective in T2D and is recognized as a key pathophysiological factor contributing to glucose intolerance [9]. The discovery of GLP-1 and GIP as incretins led to the hypothesis that they could be harnessed for glycaemic control. Early research found that while the insulinotropic actions of GIP upon exogenous administration were diminished in T2D, GLP-1 retained its ability to stimulate insulin secretion [10]. Clinical proof-of-concept emerged when a 4-hour GLP-1 infusion at supraphysiological levels normalized glucose in patients with long-standing T2D, increased insulin section and exhibited a glucose-dependent mode of action, minimizing hypoglycaemia risk [8]. Additionally, GLP-1 lowers glucose by suppressing (postprandial) glucagon secretion and slowing gastric emptying, reducing the rate of glucose absorption. However, native GLP-1 has a very short half-life (<2 minutes) due to rapid degradation by dipeptidyl peptidase-4 (DPP-4), limiting its clinical utility. This led to two pharmacological strategies for extending the glucose-lowering effect of GLP-1: GLP-1RAs, which resist DPP-4 degradation, and DPP-4 inhibitors, which prevent endogenous incretin inactivation. This review focuses on GLP-1RAs; the efficacy, safety and cardiorenal effects of DPP-4 inhibitors are reviewed elsewhere [11].

### GLP-1RAs for glucose management

In 1990, scientists discovered exendin-4 in Gila monster saliva, noting its 53% homology with human GLP-1 and its binding to pancreatic GLP-1R [11, 12]. This led to synthetic exendin-4 (known as exenatide), which is resistant to DPP-4 inactivation and effective in lowering HbA1c in T2D, gaining clinical approval in 2005 as the first GLP-1RA, administered twice daily. To extend the half-life, modified GLP-1RAs were developed, using large carrier molecules to reduce kidney clearance or absorption-delaying chemicals, enabling once-daily or once-weekly subcutaneous formulations. Since injections may hinder adherence for some patients, oral options were explored. Oral semaglutide, requiring strict fasting, was approved first, while non-peptide GLP-1RAs like orforglipron, without fasting restrictions, are under evaluation [13]. GLP-1RAs have evolved not only in their dosing frequency and administration routes, but also in efficacy. Early compounds reduced HbA1c by ≈1.0%, while newer agents achieve ≈1.5% reductions versus placebo, with efficacy also depending on dose, baseline HbA1c and background therapy. Dulaglutide and semaglutide are the most widely used and effective GLP-1RAs for glucose control in T2D, supported by extensive phase 3 trials, including head-to-head studies. In SUSTAIN 7 (NCT02648204), 1.0 mg semaglutide showed superior glycaemic control and weight reduction over dulaglutide, with similar safety profiles, in 1201 metformin-treated T2D patients over 40 weeks [14]. Recently, higher doses of dulaglutide (4.5 mg) and semaglutide (oral 25–50 mg; subcutaneous 2.0 mg) have gained approval, demonstrating superior HbA1c reduction and greater weight loss versus ‘traditional’ doses. Liraglutide, semaglutide and dulaglutide do not require dose adjustments in kidney impairment; however, caution is recommended in severe CKD due to limited clinical trial data [11].

### GLP-1RAs for weight management

The use of GLP-1-treatment for obesity originated from early T2D trials, where modest but consistent weight loss (2–5%) was observed. These findings aligned with research showing that GLP-1R activation promotes satiety and fullness and reduces appetite, hunger and food intake. While delayed gastric emptying and occasional nausea contribute to weight loss, their effects are minor and often temporary. Primarily, GLP-1 promotes satiety by directly stimulating GLP-1R in reward-related brain areas and through vagal afferents [15]. GLP-1RA-induced weight loss in humans results mainly from reduced food intake, while in rodents they activate brown fat and increase energy expenditure via the sympathetic nervous system.

The first GLP-1-RA approved for obesity treatment was liraglutide 3.0 mg daily in 2014. In the SCALE – Obesity and Prediabetes trial (NCT01272219) [16], among 3731 non-diabetic patients (mean BMI ≈38 kg/m^2^), liraglutide 3.0 mg combined with lifestyle modifications led to an 8.4-kg weight loss after 56 weeks compared with 2.8 kg with placebo. Additionally, 63% and 33% of liraglutide-treated individuals lost >5% and >10% of their body weight, respectively. Interest in GLP-1RAs for weight loss surged with the 2022 approval of semaglutide 2.4 mg weekly. The phase 3 STEP trials demonstrated its sustainable efficacy, showing weight loss of 6.2% in T2D over 68 weeks [17] and 10.3–14.9% in non-diabetics over 68–104 weeks versus placebo [18]. Consistent with other weight loss interventions, patients with T2D exhibit attenuated weight loss. Semaglutide 2.4 mg proved more efficacious than liraglutide 3.0 mg in non-diabetic individuals with overweight/obesity (−15.8% versus −6.4% at 68 weeks) [19]. Although long-term data on GLP-1RAs for weight management are limited, STEP 5 reported a sustained placebo-corrected 12.4% weight loss after 104 weeks with semaglutide, with 61.8% of participants losing >10% and more than a third exceeding 20% weight loss [20]. Semaglutide 1 mg (approved for glucose lowering in T2D) also yielded clinically meaningful weight-loss effects, albeit to a lesser degree than the dose of 2.4 mg (−6.99% versus −9.64%) [17]. However, the higher cost of the 2.4-mg dose led to a surge in off-label purchases and use of the 1.0 mg formulation, causing shortages for T2D patients. Top-line results of clinical trials assessing even higher semaglutide doses (7.2 mg weekly) in individuals with and without T2D [e.g. STEP-UP (NCT05646706)] indicate superior weight loss compared with the 2.4-mg dose (20.7% versus 17.5%), with a similar safety and tolerability profile. Since nearly half (49.1%) of adults with reduced estimated glomerular filtration rate (eGFR) have obesity—making it a more prevalent comorbidity in CKD than diabetes—and kidney impairment may limit some obesity treatment options, GLP-1RAs could be a valuable addition for this population [21].

## ‘OFF-TARGET’ EFFECTS OF GLP-1RAS

### GLP-1 actions beyond the pancreas and brain

GLP-1 actions are mainly transduced by the GLP-1R, initially identified in pancreatic β cells. However, GLP-1R expression extends to various other tissues, including the central and enteric/peripheral nervous system, lung, immune cells, heart, vessels and kidneys [11]. This widespread distribution prompted researchers to examine GLP-1 actions beyond glucose metabolism and appetite regulation. From a systems biology perspective, the gut plays a central role in sustaining normal organ functions (particularly postprandially) and can contribute to disease pathogenesis. Gut-derived molecules like GLP-1 communicate across multiple interorgan axes, including gut–brain, gut–gut/liver, gut–bone, gut–heart and gut–kidney pathways [11, 21]. For example, GLP-1 facilitates digestion and contributes to energy homeostasis by slowing the gastric emptying rate and small bowel peristalsis, exocrine secretion of bile acids, digestive enzymes and suppression of endogenous glucose production [22]. Additionally, GLP-1 helps regulate nutrient distribution and postprandial energy storage by recruiting microvasculature to peripheral tissues [23]. Moreover, GLP-1 influences water/electrolyte balance (e.g. sodium) by affecting thirst, intestinal transport and kidney functions (excretion/reabsorption) [11].

### (In)direct actions on the cardiorenal system

Since glycaemic and obesity management are central to reducing microvascular/macrovascular risk, it was hypothesized—even before the cardiovascular outcome trials (CVOTs)—that glucose- and weight-lowering effects and other ‘pleiotropic effects’ of GLP-1RAs might improve outcomes. These benefits were thought to arise through both indirect effects on cardiorenal risk factors and direct actions on the heart, vessels and kidney. GLP-1RAs increase heart rate but modestly lower BP, particularly in hypertensive individuals, reducing systolic BP by ≈2–3 mmHg [24]. Notably, this effect is not dose dependent and is partially independent of weight loss. GLP-1RAs improve lipids, particularly postprandial, with modest low-density lipoprotein (LDL)-cholesterol reduction and strong suppression of chylomicron secretion and postprandial hyperlipidaemia, even with statin use. GLP-1RAs also exhibit anti-inflammatory and antifibrotic effects within the cardiorenal system and are associated with reduced C-reactive protein and other inflammatory biomarkers, correlating with improved insulin sensitivity rather than HbA1c or weight changes [25]. Beyond fasting benefits, GLP-1RAs mitigate postprandial inflammation, oxidative stress, endothelial dysfunction, thrombogenicity, endotoxemia and altered blood/lymphatic flow [26]. Finally, GLP-1RAs were suggested to also influence glomerular hyperfiltration, albeit to a lesser extent compared with SGLT2is. In normal physiology, kidney blood flow and GFR are transiently increased after a meal, which has the potential to increase haemodynamic stress, particularly in diabetic kidney disease (DKD). Acute infusion with exenatide results in kidney afferent vasodilatation and hyperfiltration in overweight males [27], mediated in part by nitric oxide. However, dedicated mechanistic studies have failed to show significant effects of GLP-1RA infusion treatment on inulin/pulmonary arterial hypertension kidney haemodynamics in T2D patients with normal kidney function in fasting or postprandial states [11]. While SGLT2is enhance cortical (and reduce medullary) kidney oxygenation in DKD, exenatide showed no similar effects [28]. One study found that GLP-1 infusion preserved kidney oxygenation during sodium chloride loading, possibly by enhancing kidney perfusion [29].

## EFFECTS ON CV OUTCOMES

### CVOTs in T2D

Cardioprotective effects of GLP-1RAs were demonstrated in CVOTs in T2D over the past decade. Initially designed to establish CV safety per 2008 US Food and Drug Administration requirements, these trials unexpectedly revealed benefits across the drug class. Several long-acting GLP-1RAs, including liraglutide, semaglutide and dulaglutide, reduced (three-point) major adverse cardiovascular events (MACE) in high-risk T2D patients (Table 1). However, some heterogeneity was observed, as a reduction in stroke, myocardial infarction and CV death were noted in some but not all CVOTs. Most participants in GLP-1RA CVOTs had established CVD (≥70%) and were predominantly male (60–70%). However, the REWIND trial (NCT01394952) included a lower percentage of patients with prior CV events (31.5%) and a higher proportion of women (46.3%) and showed that dulaglutide reduced MACE, suggesting a role for GLP-1RAs in primary prevention in T2D. Similar trends were seen with semaglutide in post hoc analyses of PIONEER 6 (NCT02692716) and SUSTAIN-6 (NCT01720446). GLP-1RA in T2D also reduced all-cause mortality by ≈12%, even with concomitant antiplatelet, antihypertensive and lipid-lowering drugs.

**Table 1:** Cardiorenal outcomes with GLP-1RAs versus placebo (standard of care) in T2D and obesity.

						Primary composite CV outcome*		Composite kidney outcome (A)		Worsening of kidney function (B)	
Trial	Study drug	Population	Patients, *n*	Patients with CV disease, %	Follow-up (years)	HR (95% CI)	Event rate/100 patient-years	HR (95% CI)	Event rate/100 patient-years	HR (95% CI)	Event rate/100 patient-years
**Cardiovascular safety trials in T2D**											
ELIXA (2016)	Lixisenatide QD sc	T2D	6068	100	2.1	1.02 (0.89–1.17)	PBO: 6.3 GLP-1: 6.2	0.44 (0.11–1.73)	PBO: 0.1 GLP-1: 0.1	1.16 (0.74–1.83)	PBO: 0.5 GLP-1: 0.6
LEADER (2016)	Liraglutide QD sc	T2D	9340	81	3.8	**0.87 (0.78–0.97)**	PBO: 3.9 GLP-1: 3.4	0.86 (0.70–1.05)	PBO: 1.2 GLP-1: 1.0	**0.80 (0.64–0.99)**	PBO: 1.0 GLP-1: 0.8
SUSTAIN-6 (2016)	Semaglutide QW sc	T2D	3297	60	2.1	**0.74 (0.58–0.95)**	PBO: 4.2 GLP-1: 3.1	1.09 (0.75–1.58)	PBO: 1.6 GLP-1: 1.7	0.90 (0.57–1.43)	PBO: 1.2 GLP-1: 1.0
EXSCEL (2017)	Exenatide QW sc	T2D	14 752	73.1	3.2	0.91 (0.83–1.00)	PBO: 3.8 GLP-1: 3.6	0.88 (0.74–1.05)	PBO: 1.3 GLP-1: 1.2	0.90 (0.75–1.07)	PBO: 1.3 GLP-1: 1.2
Harmony (2018)	Albiglutide QW sc	T2D	9463	100	1.6	**0.78 (0.68–0.90)**	PBO: 5.7 GLP-1: 4.5	2.00 (0.37–10.87)	PBO: 0.0 GLP-1: 0.1	NR	
REWIND (2019)	Dulaglutide QW sc	T2D	9901	31.5	5.4	**0.88 (0.79–0.99)**	PBO: 2.5 GLP-1: 2.2	**0.61 (0.46–0.80)**	PBO: 0.5 GLP-1: 0.3	**0.56 (0.41–0.76)**	PBO: 0.4 GLP-1: 0.2
PIONEER-6 (2019)	Semaglutide QD oral	T2D	3183	84.7	1.3	0.79 (0.57–1.11)	PBO: 3.7 GLP-1: 2.9	NR		0.59 (0.31–1.12)	PBO: 1.2 GLP-1: 0.7
AMPLITUDE-O (2021)	Efpeglenatide QD sc	T2D	4076	89.6	1.8	**0.73 (0.58–0.92)**	PBO: 5.1 GLP-1: 3.9	0.91 (0.20–4.19)	PBO: 0.1 GLP-1: 0.2	1.50 (0.30–7.45)	PBO: 0.1 GLP-1: 0.1
FREEDOM-CVO (2022)	Exenatide sc-infusion	T2D	4156	76.0	1.4	1.21 (0.90–1.63)	PBO: 2.4 GLP-1: 2.9	NR		NR	
											
SOUL (2025)	Semaglutide QD oral	T2D	9650	100	4.1	**0.86 (0.77–0.96)**	PBO: 3.7 GLP-1: 3.1	0.86 (0.66–1.10)	PBO: 0.7 GLP-1: 0.6	NR	
**Renal outcome trial in T2D**											
FLOW (2024)	Semaglutide QW sc	T2D	3533	22.9	3.4	**0.79 (0.66–0.94)**	PBO: 4.2 GLP-1: 3.5	**0.79 (0.66–0.94)**	PBO: 4.3 GLP-1: 3.6	**0.73 (0.59–0.90)**	PBO: 3.5 GLP-1: 2.7
**Cardiovascular outcome trials in obesity**											
SELECT (2023)	Semaglutide QD sc	Obesity	17 604	100	3.3	**0.80 (0.72–0.90)**	PBO: 2.4 GLP-1: 2.0	0.62 (0.33–1.16)	PBO: 0.1 GLP-1: 0.1	0.57 (0.28–1.17)	PBO: 0.0 GLP-1: 0.1

NR: not reported; sc: subcutaneous; SCr: serum creatinine; QD: once daily; QW: once weekly.

Significant values in bold.

Data adapted from the original papers and Badve *et al.* [76].

*The primary endpoint in these trials was the first occurrence of a 3-point MACE, consisting of cardiovascular death, myocardial infarction and stroke. (A) Composite kidney outcome. In the FLOW, LEADER, REWIND, SUSTAIN-6, SELECT, SOUL and AMPLITUDE-O trials, this outcome was a sustained reduction in eGFR by ≥50%, kidney failure (persistent eGFR <15 ml/min/1.73 m^2^ or initiation of kidney replacement therapy) or death due to kidney failure. In the EXSCEL trial, this outcome was a sustained reduction in eGFR by ≥40%, kidney failure (persistent eGFR <15 ml/min/1.73 m^2^ or initiation of kidney replacement therapy) or death due to kidney failure. In the ELIXA and Harmony trials, this outcome was kidney replacement therapy. (B) Worsening of kidney function. In the FLOW, LEADER, REWIND, SUSTAIN-6, PIONEER 6, SELECT and AMPLITUDE-O trials, this outcome was a sustained decline in eGFR by ≥50%. In the EXSCEL trial, this outcome was a sustained decline in eGFR by >40%. In the ELIXA trial, this outcome was a doubling of serum creatinine.

Additionally, randomized controlled trial (RCT) and real-world data suggest additive CV benefits when combined with SGLT2i. While individual CVOTs showed mixed results on HF hospitalization, pooled data from >60 000 T2D patients demonstrated an 11% reduction [30]. Furthermore, the STEP-HFpEF DM trial (NCT04916470) found that semaglutide 2.4 mg improved HF-related symptoms and physical function in T2D patients with heart failure with preserved ejection fraction (HFpEF) and obesity [31].

### People with overweight or obesity

In the SCALE trial, including 5908 participants with overweight/obesity, the CV event rate with liraglutide 3.0 mg was low (1.54 events/1000 person-years). Additionally, retrospective adjudication of events in two of the trials limited the ability to accurately determine its CV benefit compared with comparators, despite a favourable trend {hazard ratio [HR] 0.42 [95% confidence interval (CI) 0.17–1.08]}. A 2022 meta-analysis of 4582 participants in STEP1–4 assessed semaglutide 2.4 mg in obesity and found a reduced risk of pooled major or minor CV disorders [HR 0.70 (92% CI 0.57–0.87)], although MACE were too few for conclusions [32]. The dedicated SELECT trial then evaluated semaglutide 2.4 mg in 17 604 overweight/obese patients (BMI 33.3 kg/m^2^) with CVD but without T2D (64% pre-diabetes). Over 39.8 months, semaglutide decreased 3-point MACE by 20% (primary outcome; 95% CI 0.72–0.90), ultimately confirming its CV benefit in this population [33]. In the recent SCORE real-world analysis (NCT06874751), semaglutide 2.4 mg was associated with a 57% lower risk of 3-point MACE compared with non-users (95% CI 0.31–0.61). Also, in STEP-HFpEF, semaglutide improved symptoms, physical limitations and exercise function in 529 obese non-diabetic patients with HFpEF [34].

### Mediation analyses for CV outcomes

The longer time periods, typically >1 year, required to observe reduced MACE in GLP-1RA CVOTs compared with the quicker effects seen in SGLT2i CVOTs likely reflect a reduction in atherosclerosis rather than acute haemodynamic effects. Neither HbA1c reduction nor weight loss fully accounts for the CV benefits observed with GLP-1RA. While a correlation exists between HbA1c reduction and MACE ratios in individual GLP-1RA CVOTs [35], this does not fully explain the CV benefit, especially considering the effect in non-diabetic individuals. Mediation analyses from the LEADER (NCT01179048) and REWIND trials suggests that only half of the effect in T2D is due to HbA1c lowering [36]. In recent analyses, weight loss linked to GLP-1RAs correlated with improved CV outcomes [26]. In STEP-HFpEF, benefits were seen across various BMIs but were directly proportionate to the extent of weight loss [37]. However, in HARMONY Outcomes (NCT02465515), albiglutide reduced MACE by 22% with only modest weight loss (0.83 kg), suggesting that GLP-1RA CV benefits are not solely dependent on HbA1c lowering, weight loss or the presence of T2D.

## EFFECTS ON KIDNEY OUTCOMES

### From CVOTs to FLOW

Evidence suggests GLP-1RAs also have kidney-protective effects (Table 1). Phase 3 trials in T2D documented albuminuria reductions [38], yet data were heterogenous, and interventions generally did not affect eGFR. It is important to highlight that these studies were generally short term and included populations selected for diabetes and/or obesity, resulting in low kidney event rates (secondary or exploratory outcomes) and were consequently underpowered to detect significant changes in eGFR or clinical kidney outcomes. In the SCALE – Diabetes trial (NCT01272232), liraglutide 3.0 mg decreased urine albumin:creatinine ratio (UACR) in a dose-dependent manner [39] and dulaglutide, in 6005 T2D patients, also lowered UACR versus placebo, insulin glargine or other active comparators in an integrated data analysis of nine trials [40]. However, the LIRA-RENAL trial (NCT01620489) in DKD patients showed no significant UACR changes with liraglutide [41].

Secondary/post hoc analyses of CVOTs showed that GLP-1RAs reduce new-onset macroalbuminuria (typically included as a secondary/exploratory endpoint), independent of HbA1c changes [11, 42]. While CVOTs consistently demonstrate albuminuria-lowering effects of GLP-1RA, the relatively short duration of CVOTs, their focus on CV outcomes and the exclusion of patients with severe kidney insufficiency (possibly leading to reduced drug tolerability) resulted in a low incidence of kidney events (<1%; mean 0.61 events/100 patient-years across all CVOTs; Table 1) and limited power to identify hard kidney benefits. In line with this, CVOTs have generally been limited in demonstrating renoprotection with GLP-1RAs on clinical kidney outcomes; however, we emphasize that the albuminuria-lowering effect remains a strong and reasonably likely surrogate for clinical kidney outcomes that should not be overlooked.

Pooled analysis of the SUSTAIN-6 and LEADER trials [43] showed that semaglutide and liraglutide slowed eGFR decline and reduced the risk of sustained eGFR reductions (≥40% and ≥50%) compared with placebo, especially in CKD. In the REWIND trial, enriched with CKD patients, dulaglutide decreased a composite kidney outcome (that did not include albuminuria) by 28% (95% CI 0.58–0.88) and decreased the incidence of sustained eGFR decline of ≥40% (HR 0.70) or ≥50% (HR 0.56), yet event rates were still low [44]. In contrast, the EXSCEL trial (NCT01144338) showed no significant kidney benefits with exenatide, with favourable effects only in subgroups on eGFR slope and albuminuria [45]. In the AWARD-7 trial (NCT01621178), dulaglutide slowed the eGFR decline and reduced the risk of a ≥40% decrease (HR 0.45) in 577 T2D patients with moderate–severe CKD compared with insulin glargine. Also, dulaglutide reduced a relevant composite kidney outcome [i.e. ≥40% eGFR decline, end-stage renal disease (ESRD) or kidney death] in participants with severely increased albuminuria by 75% (7% versus 22%; 95% CI 0.10–0.68) [46]. GLP-1RA-induced effects on kidney outcomes in T2D were more definitively confirmed by the recent dedicated FLOW trial (NCT03819153) [47]. Enrolling 3533 patients with long-standing T2D and CKD (68% classified as very high risk), FLOW treated participants with 1.0 mg semaglutide or placebo on a background of standard of care for 3.4 years. Stopped early due to efficacy, semaglutide reduced the primary kidney outcome (persistent eGFR decline ≥50%, ESRD or kidney- or CV-related death) by 24% (95% CI 0.66–0.88), with similar results found for all kidney-specific components. All secondary outcomes favoured semaglutide, including a slower eGFR decline (1.16 ml/min/1.73 m^2^) and a reduced rate of MACE and all-cause mortality (by 18% and 20%, respectively). Semaglutide’s benefits on cardiorenal outcomes were consistent regardless of background SGLT2i use [48], although baseline use was low (15.6%), reflecting standard practice when the FLOW trial was initiated in 2019.

Semaglutide may also benefit kidney outcomes in overweight/obesity without T2D. In a secondary analysis of SELECT, semaglutide reduced the incidence of a prespecified composite kidney endpoint by 22% (95% CI 0.63–0.96). At 104 weeks, treatment benefit for eGFR was 0.75 ml/min/1.73 m^2^ overall and 2.19 ml/min/1.73 m^2^ in patients with eGFR <60 ml/min/1.73 m^2^ [49]. In the SMART trial (NCT04889183), semaglutide reduced UACR by 52.1% in 101 non-diabetic patients with overweight/obesity and CKD (mostly due to chronic glomerulonephritis or hypertension) [50].

### Mediation analyses for kidney outcomes

The significant impact of GLP-1RAs on new-onset macroalbuminuria in some CVOTs may largely reflect the differences in glucose lowering between treatment arms [11]. A meta-regression analysis of CVOTs with kidney outcomes showed that a 1.0% reduction in HbA1c was linked to a 35% decrease in the log-HR for kidney outcomes [51], while weight loss had no association. In the LEADER and SUSTAIN-6 trials, HbA1c accounted for 25–26% of kidney effects, while systolic BP contributed 9–22% (with little or no mediation by body weight). Benefits in obesity without T2D (SELECT and SMART trials) also suggest additional renoprotective pathways beyond HbA1c.

## SAFETY AND TOLERABILITY OF GLP-1RAS

The most common adverse effects (AEs) of GLP-1RAs are gastrointestinal, such as nausea, diarrhoea and vomiting. Up to 50% may experience this, particularly during dose initiation/escalation. Although these are typically mild to moderate and usually resolve over 4–8 weeks, they can be dose-limiting and may prompt discontinuation (≈12% in phase 3 trials). Severe, persistent symptoms may limit fluid intake and lead to dehydration and acute kidney injury [11]. The risk of hypoglycaemia is generally low in individuals with T2D or obesity, except when combined with insulin/sulfonylureas for glucose lowering.

Despite concerns regarding the long-term safety of GLP-1RAs due to the extrapancreatic expression of GLP-1Rs, they have been in clinical use for 2 decades, with mild AEs reported in long-term studies. While there were initial worries about pancreatitis and pancreatic cancer, clinical trials and real-world data have shown no imbalance in pancreatic AEs [11]. GLP-1RAs have been linked to thyroid C cell tumours in rodents, but no increased risk is observed in humans. Rapid weight loss by GLP-1RA may increase the number of gallbladder-related AEs. Also, one semaglutide CVOT reported an increase in retinopathy events, likely due to rapid glucose lowering in patients with active retinopathy [52]. Ongoing trials, such as FOCUS (NCT03811561), are studying semaglutide's safety in T2D patients with diabetic retinopathy (results expected in 2027). Until then, screening for retinopathy before initiating semaglutide and discussing risk:benefit ratios is recommended.

Concerns about lean body mass (LBM) loss due to GLP-1RA-induced weight loss, especially in sarcopenia, have emerged. However, body composition analyses in T2D showed no consistent evidence of disproportionate LBM loss or impaired muscle strength. In the STEP-1 trial, while semaglutide reduced dual-energy X-ray absorptiometry–measured total LBM, the proportion of LBM relative to total body mass increased [53]. Ongoing trials, including those combining semaglutide with bimagrumab (NCT05616013), aim to explore methods to preserve LBM and muscle strength during GLP-1RA-induced weight loss. It is important to note that some nutritional strategies, such as high protein (1.2–2 g/kg/day) and carbohydrate-restricted diets, are currently recommended to help maintain muscle mass. Although limited evidence exists regarding optimal protein/carbohydrate intake, specifically in patients with CKD and diabetes or obesity, these approaches may theoretically raise safety concerns due to their potential to exacerbate glomerular hyperfiltration or induce ketosis, especially when combined with SGLT2 inhibitors [54]. Research will determine if functional muscle loss becomes an issue requiring additional interventions beyond standard care, including individualized dietary advice and exercise, with careful consideration of renal and metabolic safety in this population.

## BEYOND GLP-1RAS: MULTIRECEPTOR AGONISTS

### From physiology to pharmacology

As our understanding of incretin/enteroendocrine biology matured, recent drug development efforts have focused on combining GLP-1RA with other hormonal stimuli. Researchers have engineered unimolecular agonists that interact with multiple enteropancreatic hormone receptors to enhance efficacy and clinical outcomes. This strategy mirrors normal gut physiology, where enteroendocrine cells secrete dozens of hormones involved in glucoregulatory/anorectic functions. The success of bariatric (metabolic) surgeries like Roux-en-Y gastric bypass, which increases circulating GLP-1 and other gut hormones, highlights the potential of these therapies in reducing appetite, promoting weight loss and improving glucose control. These benefits are thought to stem from neuroendocrine input rather than its restrictive nature. Gut hormones also play a role in weight regain after significant weight loss by lowering the metabolic rate and increasing hunger signals. Developing unimolecular agonists presents challenges, requiring careful attention to molecular ratios and potential AEs. Several enteropancreatic hormones, such as GIP, glucagon, amylin and peptide YY, are being investigated for their roles in enhancing GLP-1R agonism in metabolic diseases. Tirzepatide, a dual GIP–GLP-1R co-agonist approved for T2D and obesity management demonstrates potential additive/synergistic effects between two hormones (Supplementary Fig. 1) and has achieved ≥20% weight loss in non-T2D [55]. Other molecules in late-phase trials may offer similar benefits and efficacy that may that approach Roux-en-Y gastric bypass.

### Tirzepatide: a GIP–GLP-1 co-agonist

Initially GIP receptor (GIP-R) agonism for obesity/T2D was met with scepticism due to GIP's diminished insulinotropic effect in T2D and evidence suggesting GIP-R antagonism could also improve energy/glucose metabolism. However, tirzepatide has become the most effective GLP-1-based drug to date for T2D and obesity. In the SURPASS trials, tirzepatide reduced HbA1c by 1.9–2.6% across trials, with 40–69% of T2D participants losing ≥10% body weight at higher doses. In the SURPASS-2 trial (NCT03987919), tirzepatide outperformed semaglutide 1.0 mg in both HbA1c and weight loss [56]. The SURMOUNT program showed 16–22.5% weight loss in non-DM obese individuals, without signs of plateau. Gastrointestinal AEs with tirzepatide in the SURMOUNT program appear lower than those with semaglutide in the STEP trials, possibly due to the role of GIP in reducing aversive central responses. The exact molecular mechanisms of tirzepatide on weight/glucose remain uncertain, with the role of GIP-R activation remaining unclear. Clinical evaluation of AMG133, a GIP-R antagonist, underscores the intrigue of GIP-targeted therapies, demonstrating weight loss of up to 14.5% in phase 1 and now moving to phase 2 (NCT05669599).

Development of tirzepatide reignited interest in the cardiorenal actions of GIP. A prespecified meta-analysis of CV events in 7215 T2D patients from the SURPASS trials showed CV safety, with an HR of 0.80 (95% CI 0.57–1.11) for 4-point MACE and 0.80 (95% CI 0.51–1.25) for all-cause mortality [57]. In the SUMMIT trial (NCT04847557), tirzepatide reduced the risk of death from CV causes or worsening HF by 38% and substantially improved HF symptoms in 731 patients with HFpEF and obesity [58]. Regarding kidney outcomes, tirzepatide reduced new-onset macroalbuminuria by 59%, and slowed eGFR decline by 2.2 ml/min/1.73 m^2^/year compared with glargine in a post hoc analysis of SURPASS-4 (NCT03730662) [59]. Additionally, tirzepatide decreased UACR compared with other comparators (including semaglutide) in a pooled analysis of the SURPASS trials [60]. The CV safety of tirzepatide is being further evaluated in T2D patients with confirmed CVD (SURPASS CVOT; versus dulaglutide) and in individuals with obesity [SURMOUNT-MMO (NCT05556512)], with kidney outcomes assessed in TREASURE-CKD (NCT05536804) (Table 2).

**Table 2:** Ongoing relevant trials of GLP-1-based drugs on cardiorenal outcomes.

Trial (NCT number)	Drug formulation	Interventions	Population	Enrolment	Primary endpoint	Kidney endpoint	Study completion (estimated)
GLP-1 receptor agonists							
ADJUST-T1D (NCT05537233)	GLP-1	Semaglutide versus placebo	T1D + BMI ≥30 kg/m^2^	115	CGM, measured time in range	Change in eGFR and UACR	8/2024
REMODEL (NCT04865770)	GLP-1	Semaglutide versus placebo	T2D + CKD	106	Kidney oxygenation	Change in eGFR and UACR	11/2024
ACHIEVE-4 (NCT05803421)	GLP-1 (oral)	Orforglipron versus insulin glargine	T2D + CVD/CKD	2620	3-point MACE	Not specified	9/2025
RT1D (NCT05822609)	GLP-1	Semaglutide versus placebo	T1D + CKD	60	Kidney oxygenation	Change in eGFR and UACR	6/2026
T1-DISCO (NCT05819138)	GLP-1	Semaglutide versus placebo	T1D	60	Pulse wave velocity	Renal vascular resistance	12/2026
ASCEND PLUS (NCT05441267)	GLP-1 (oral)	Semaglutide versus placebo	T2D	20 000	5-point MACE	Not specified	8/2028
PRECIDENTD (NCT05390892)	GLP-1	GLP-1RA versus SGLT2i	T2D (70% secondary prevention cohort)	6000	4-point MACE, 2-point kidney composite and mortality	Not specified	3/2029
GLP-1/GIP							
SURPASS-CVOT (NCT04255433)	GIP/GLP-1	Tirzepatide versus dulaglutide	T2D + CVD	13 299	3-point MACE	Secondary: change in UACR, new or worsening nephropathy	6/2025
TREASURE-CKD (NCT05536804)	GIP/GLP-1	Tirzepatide versus placebo	BMI ≥27 kg/m^2^ + CKD	140	Kidney oxygenation (BOLD-MRI)	Secondary: change in renal sinus fat, kidney blood flow, GFR (iohexol), 24-h UAE, UACR	10/2026
SURMOUNT-MMO (NCT05556512)	GIP/GLP-1	Tirzepatide versus placebo	BMI ≥27 kg/m^2^ + CVD/risk factors	15 374	5-point MACE	Secondary: eGFR slope and composite of kidney endpoint (≥40% eGFR decline, ESRD or kidney death)	10/2027
Other							
CagriSema phase 2 (NCT06131372)	GLP-1/amylin	CagriSema versus placebo	T2D + CKD	618	Change in UACR	Secondary: change in eGFR	12/2025
SYNCHRONIZE-CVOT (NCT06077864)	GLP-1/glucagon	Survodutide versus placebo	BMI ≥27 kg/m^2^ + CVD/CKD	5550	5-point MACE	Not specified	7/2026
REDEFINE-3 (NCT05669755)	GLP-1/amylin	CagriSema versus placebo	BMI ≥25 kg/m^2^ + CVD	7000	3-point MACE	Secondary: composite (≥40% reduction eGFRcr, persistent eGFRcr <15, dialysis, kidney/CV death)	10/2027
TRIUMPH-OUTCOMES (NCT06383390)	GIP/GLP-1/glucagon	Retatrutide versus placebo	BMI ≥27 kg/m^2^ + CVD/CKD	10 000	4-point MACE, composite of ESRD, ≥40% eGFR and CV/kidney death	Secondary: composite kidney endpoint (≥40% eGFR decline, ESRD or renal death), change in UAC	2/2029

BOLD-MRI: blood oxygen level–dependent magnetic resonance imaging; CGM: continuous glucose monitor; eGFRcr: estimated glomerular filtration rate with creatinine; T1D: type 1 diabetes mellitus; UAE: urinary albumin excretion.

### Other dual and triple agonists

Glucagon-receptor agonism reduces food intake and increases energy expenditure, supporting weight loss. Combined with GLP-1R agonism, this synergy may not only enhance weight loss but may also mitigate glucagon-induced hyperglycaemia. It also improves lipid metabolism and hepatic fatty acid oxidation, which may benefit conditions like MASLD/metabolic dysfunction-associated steatohepatitis (MASH). Survodutide, a GLP-1/glucagon co-agonist currently in phase 3 trials (SYNCHRONIZE) for obesity treatment, achieved up to 18.7% weight loss after 46 weeks of weekly dosing (0.6–4.8 mg) in phase 2 [58], although gastrointestinal AEs led to discontinuation rates of 20–29%. SYNCHRONIZE – CVOT (NCT06077864) is assessing its CV safety in 5550 individuals (expected results in 2026; Table 2). Other candidates in late-stage clinical testing for metabolic diseases include mazdutide, pemvidutide and efinopegdutide.

Given the success of tirzepatide and GLP-1/glucagon-RAs, a triple agonist targeting GLP-1, GIP and glucagon could trigger even more weight loss and better glycaemic control. Retatrutide, a triple GLP-1/GIP/glucagon-RA, has demonstrated greater efficacy than tirzepatide in preclinical models by increasing energy expenditure and reducing calorie intake. In phase 2 for obesity [61], retatrutide (1–12 mg) induced dose-dependent weight loss, reaching 24.2% at 48 weeks versus 2.1% with placebo. At week 48, 36–48% of individuals receiving the highest two doses achieved ≥25% weight loss, along with notable improvements in lipids and BP. Phase 3 trials are assessing retatrutide's efficacy and safety in various obesity populations [including T2D, obstructive sleep apnoea (OSA) and osteoarthritis] with a 10 000-patient CVOT in overweight/obese individuals with atherosclerotic CVD and/or CKD (Table 2).

In addition to GIP/glucagon, other receptors are being explored alongside GLP-1R. One such approach involves cagrilintide, a long-acting amylin analogue, which achieved a placebo-subtracted weight loss of 7.6% at the highest dose (4.5 mg). Cagrilintide is being studied in combination with semaglutide (CagriSema), showing promise in achieving >20% weight loss and glycaemic control [62]. CagriSema is in phase 3 trials for T2D and obesity, including a head-to head study against tirzepatide (REDEFINE-4) and a dedicated CVOT (Table 2).

## CLINICAL IMPLICATIONS AND FUTURE PERSPECTIVE

### Position of GLP-1RA in clinical CKD guidelines

Current clinical guidelines recognize GLP-1RAs as cornerstone drug therapies for managing T2D with CKD. The 2025 American Diabetes Association Standards of Care [63] emphasize prioritizing drugs that reduce CV/CKD risk in patients with T2D and established ASCVD, HF or CKD, with emphasis on SGLT2is and GLP-1RAs for their proven benefits. SGLT2is are recommended for DKD patients with eGFR ≥20 ml/min/1.73 m^2^, with GLP-1RAs suggested when CV risk is a primary concern and to slow CKD progression. When selecting glucose-lowering medications, individual patient risk, as well as effects on weight, AEs, personal preferences and cost considerations should further guide therapy choices. For patients with advanced CKD (eGFR <30 ml/min/1.73 m^2^), GLP-1RAs are preferred over insulin due to their lower risk of hypoglycaemia, CV/kidney benefits and weight effects. GLP-1RAs can be used in patients with low eGFR, although dose adjustments may be required, except for liraglutide, semaglutide and dulaglutide, which do not require adjustments. Evidence from trials like FLOW is starting to position GLP-1RAs as potential fourth foundational therapies—alongside RAAS inhibitors, SGLT2is and finerenone—initially for DKD (Supplementary Fig. 2) and possibly broader use.

### Combination therapy for metabolism and CV/renoprotection

**Figure 2:**
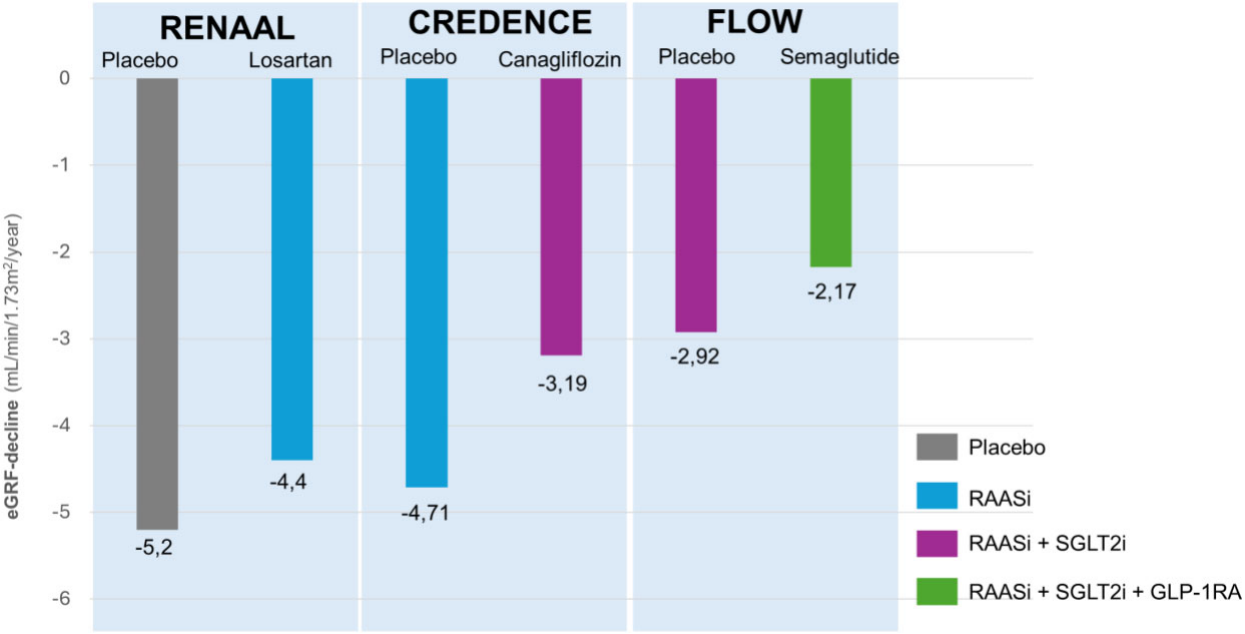
Changes in eGFR (ml/min/1.73 m^2^/year) observed in the RENAAL [64], CREDENCE [65] and FLOW [48] trials according to treatment group, illustrating the beneficial impact of combination therapy with a RAASi, SGLT2i and GLP-1RA on slope reduction in patients with T2D and CKD. RAASi: renin–angiotensin–aldosterone system inhibitor.

Many highlight the potential benefits of combining GLP-1RAs with SGLT2is, as well as with RAAS inhibitors and finerenone, for comprehensive risk reduction and addressing persistent residual risk in CV and kidney outcomes in metabolic diseases, given their distinct cardiorenal and metabolic mechanisms of action, which may be additive/synergistic. Combining data from three successive landmark trials (RENAAL [64], CREDENCE [65] and FLOW [48]) demonstrated that the sequential addition of RAAS inhibitors, SGLT2is and GLP-1RAs progressively slows the annual eGFR decline in patients with T2D and CKD (Fig. 2), underscoring the additive kidney-protective effects of multidrug therapy targeting distinct pathophysiological mechanisms. Guidelines recommend that healthcare professionals use their best judgment when deciding which medication to prescribe initially and in combination. However, initiation studies and specific combinations of renoprotective drugs have not been fully explored, leaving several questions and uncertainties. Current dedicated CV/kidney trials typically involve participants using a RAAS inhibitor. Yet, background use of SGLT2is and finerenone in CVOTs or dedicated outcome trials for GLP-1RAs remains limited, which restricts definitive post hoc conclusions on combination therapy.

The DURATION-8 trial (NCT02229396) demonstrated that combining SLGT2is with GLP1-RAs in T2D resulted in greater improvements in glucose, BP and body weight compared with either therapy alone [66]. In addition, pooled data from eight RCTs involving 1895 T2D patients showed that compared with SGLT2i or GLP-1RA monotherapy, combination therapy led to greater reductions in HbA1c (by 0.77%), fasting and postprandial glucose, body weight, LDL-cholesterol and systolic BP [67]. In the AMPLITUDE-O trial (NCT03496298), which uniquely stratified randomization by baseline or anticipated SGLT2i use and had the highest SGLT2i prevalence [*N* = 618 (15.2%)] among GLP-1RA CVOTs to date, SGLT2i did not alter efpeglenatide's kidney benefit [68]. In the FLOW trial, the effects of semaglutide on cardiorenal outcomes were consistent regardless of background SGLT2i use (15.6% at baseline, increasing to 33% by trial end, with a higher proportion of users in the placebo group compared with semaglutide) [48]. The largest meta-analysis to date, which included 12 CVOTs with 73 238 T2D patients, of whom 3065 (4.2%) were also on GLP-1RAs, confirmed that the CV/kidney benefits of SGLT2i remain consistent regardless of GLP-1RA use. Mechanistic trials in humans suggest that this combination therapy may even provide additive CV/kidney protection. The DECADE trial (NCT02004613) found that combining dapagliflozin and exenatide resulted in a greater reduction in UACR (26%) compared with dapagliflozin (22%) or exenatide (8%) alone in 20 DKD patients [69]. Similarly, the DECREASE trial (NCT03361098), which included T2D patients with obesity, showed that combining exenatide and dapagliflozin led to a greater UACR reduction at 16 weeks (40%) compared with dapagliflozin (16%) or exenatide (16%) monotherapy [70]. Additionally, combination therapy produced a larger initial decrease in cystatin C eGFR from baseline (10.4 ml/min/1.73 m^2^) than monotherapy, a change often associated with more favourable long-term kidney outcomes. Two studies examined the impact of combined SGLT2i/GLP-1RA on magnetic resonance imaging–assessed kidney function and oxygenation in T2D patients [28, 71]. Findings suggest that SGLT2i increases cortical and reduces medullary kidney oxygenation, while combination therapy with a GLP-1RA may improve kidney perfusion in the medulla. Real-world data further supports enhanced CVD protection, showing that combination SGLT2i/GLP-1RA therapy is associated with a reduction in MACE, as well as HF, compared with other glucose-lowering therapies [72]. A meta-analysis of 1604 T2D patients found that combination therapy decreased the incidence of CV events compared with active treatment or placebo [relative risk 0.19 (95% CI 0.04–0.96)] without increasing severe hypoglycaemia incidence [73]. These findings suggest that SGLT2is and GLP-1RAs provide independent complementary benefits, supporting clinical guidelines that recommend their combined use to improve CV/kidney outcomes in T2D and potentially beyond.

### Ongoing cardiorenal studies and future directions

GLP-1RAs and next-generation GLP-1-based therapies are being extensively investigated across an expanding range of metabolic and non-metabolic conditions associated with obesity and weight gain. In addition, GLP-1RAs are being studied in patients with type 1 diabetes, with or without obesity, to assess their effects on glycaemic control, weight loss, insulin sensitivity, cardiorenal outcomes [e.g. ADJUST-T1D (NCT05537233), RT1D (NCT05822609) and T1-DISCO (NCT05819138)] and safety, including risks of hypoglycaemia and ketoacidosis. While CV/kidney effects remain a primary focus of most ongoing trials (Table 2), emerging evidence also suggests broader therapeutic potential. For instance, GLP-1-based therapies have been shown to improve walking distance in patients with symptomatic peripheral artery disease [74], reduce hepatic fat accumulation and fibrosis progression in MASLD/MASH, offer potential benefits in neurodegenerative diseases such as Parkinson's and Alzheimer's, alleviate osteoarthritis pain and mitigate symptoms of OSA [75]. Additionally, ongoing research is investigating their effects on reproductive health, potential antiproliferative properties in cancer and impacts on asthma exacerbations, while anecdotal reports suggest possible influences on addictive behaviours such as smoking, alcohol use and compulsive habits [75]—although rigorous studies are needed. Given these emerging and potential indications, research should prioritize assessing their efficacy, particularly in these understudied areas, while also evaluating long-term safety and sex-specific responses and refining personalized therapeutic strategies.

## CONCLUSION

The development of GLP-1-based therapies, from exenatide 2 decades ago to advanced agents like semaglutide and tirzepatide, has significantly enhanced the outlook and clinical outcomes for patients with T2D and/or obesity and cardiorenal disease. Extensive clinical trial data and real-world evidence demonstrates their benefits, not only in reducing HbA1c and body weight, but also in glucose and weight-independent reductions in MACE, HF and CKD progression, particularly in high-risk T2D populations, with emerging data also reporting benefits in those without diabetes. Next-generation GLP-1-based therapies, including hybrid molecules also targeting GIP, glucagon and amylin, show promise in further improving metabolic and cardiorenal outcomes compared with single GLP-1R agonism, but require careful safety and efficacy evaluations. Precision medicine could help identify individuals who will benefit the most. Ongoing research, innovation and policy initiatives are crucial to realizing the full potential of GLP-1-based therapies. These efforts are key to minimizing residual cardiorenal risks worldwide and tackling other health outcomes associated with metabolic diseases in the face of the expanding epidemic.

## SEARCH STRATEGY AND SELECTION CRITERIA

We searched MEDLINE, PubMed, Google Scholar and the Cochrane Library for English-language abstracts and full-text articles published through April 2025. We focused on the cardiorenal effects of new GLP-1-based therapeutics (particularly GLP-1 and GIP, and including drug combinations with glucagon and amylin) in T2D and obesity, with particular attention paid to GLP-1RAs as monotherapy and in combination with current cardiorenoprotective therapies available or in development for T2D (SGLT2is, RAAS inhibitors and mineralocorticoid receptor antagonists). The keywords used included ‘cardiovascular disease’, ‘kidney disease’, ‘diabetic nephropathy’, ‘cardioprotection’, ‘renoprotection’, ‘type 2 diabetes’, ‘obesity’, ‘incretin-based therapy’, ‘glucagon-like peptide-1’, ‘GLP-1 receptor agonist’, ‘GIP’, ‘dual agonists’, ‘triple agonists’, ‘glucagon’ and ‘tirzepatide’. These keywords were used as single search terms and in combination. We also searched the reference list of original articles, narrative reviews, clinical guidelines and systematic reviews and meta-analyses for further relevant material. The evidence discussed in this review is restricted to clinical studies, including cohort studies, randomized controlled trials and meta-analyses of RCTs.

## Supplementary Material

gfaf110_Supplemental_File

## Data Availability

No new data were generated or analysed in support of this research.
