# Supplementary material for: GLP-1-based therapeutics for cardiorenal protection in metabolic diseases
Source: Nephrol Dial Transplant. 2025 Jun 25;41(2):207–19. doi: 10.1093/ndt/gfaf110 (PMC12855604; doi:10.1093/ndt/gfaf110)
Supplement: gfaf110_Supplemental_File [file gfaf110_supplemental_file.docx]

**Supplemental Figure 1:** Schematic Representation of the Physiological and Pharmacological Effects of Glucose-Dependent Insulinotropic Polypeptide (GIP) and Glucagon-Like Peptide-1 (GLP-1)


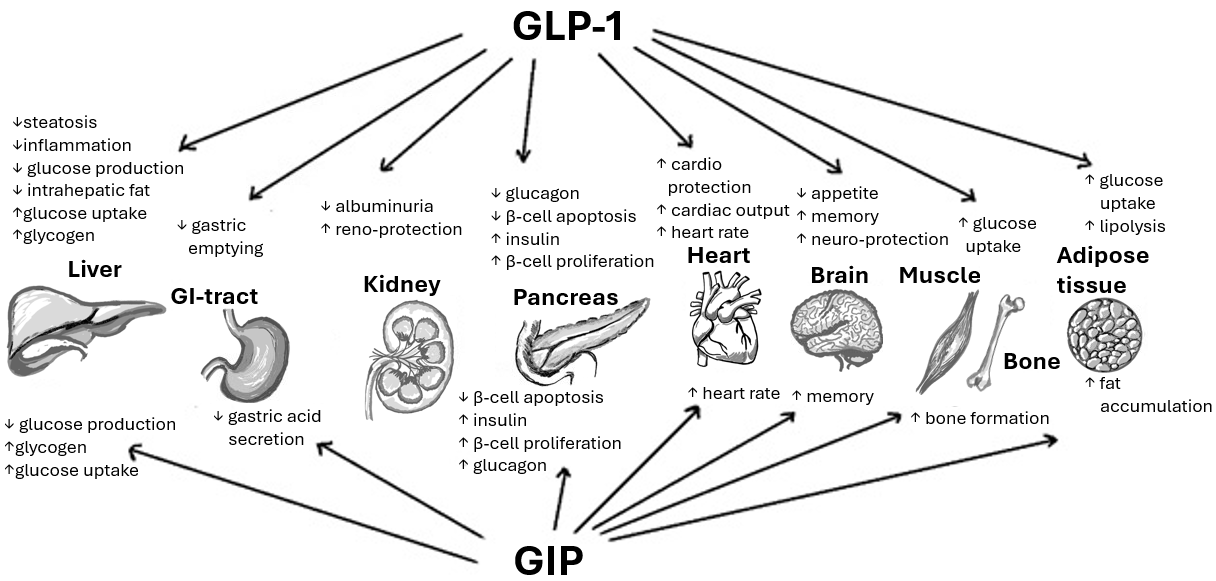


**Supplemental Figure 2:** The potential four pillars of treatment for cardiorenal protection patients with type 2 diabetes and CKD


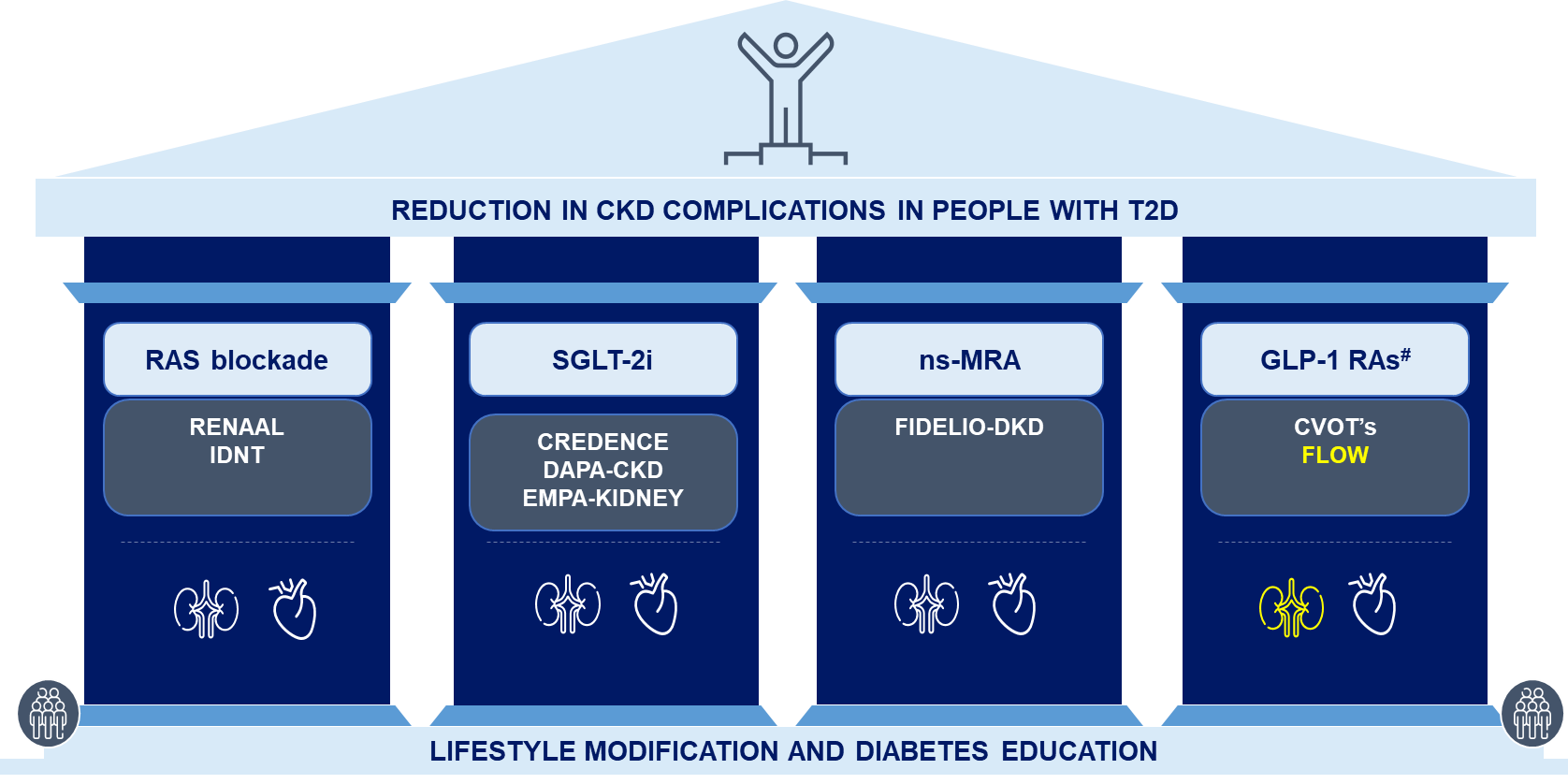


#The definitive place in the treatment of patients with T2D and CKD remains to be established. Semaglutide is the only GLP-1RA demonstrating effects in a dedicated kidney outcomes trial. Abbreviations: CKD, chronic kidney disease; CVOTs, cardiovascular outcomes trials; GLP-1 RAs, glucagon-like peptide-1 receptor agonists; ns-MRA, non-steroidal mineralocorticoid receptor antagonists; RAS, Renin–angiotensin system; SGLT-2i, sodium-glucose cotransporter-2 inhibitor; T2D, type 2 diabetes.
